# Targeting Protective Autophagy Exacerbates UV-Triggered Apoptotic Cell Death

**DOI:** 10.3390/ijms13011209

**Published:** 2012-01-20

**Authors:** Li-Hsin Chen, Pei-Ming Chu, Yi-Jang Lee, Pang-Hsien Tu, Chin-Wen Chi, Hsin-Chen Lee, Shih-Hwa Chiou

**Affiliations:** 1 Institute of Pharmacology, School of Medicine, National Yang-Ming University, Taipei 11221, Taiwan; E-Mails: jenhsinchen@gmail.com (L.-H.C.); cwchi@vghtpe.gov.tw (C.-W.C.); 2 Graduate Institute of Life Sciences, National Defense Medical Center, Taipei 11490, Taiwan; E-Mail: chupeiming@yahoo.com.tw; 3 Department of Biomedical Image and Radiological Sciences, School of Biomedical Science and Engineering, National Yang-Ming University, Taipei 11221, Taiwan; E-Mail: yjlee2@ym.edu.tw; 4 Institute of Biomedical Sciences, Academia Sinica, Taipei 11529, Taiwan; E-Mail: btu@ibms.sinica.edu.tw; 5 Department of Medical Research and Education, Taipei Veterans General Hospital, Taipei 11217, Taiwan

**Keywords:** autophagy, apoptosis, UV

## Abstract

Autophagy is activated by various stresses, including DNA damage, and previous studies of DNA damage-induced autophagy have focused on the response to chemotherapeutic drugs, ionizing radiation, and reactive oxygen species. In this study, we investigated the biological significance of autophagic response to ultraviolet (UV) irradiation in A549 and H1299 cells. Our results indicated that UV induces on-rate autophagic flux in these cells. Autophagy inhibition resulting from the knockdown of beclin-1 and Atg5 reduced cell viability and enhanced apoptosis. Moreover, we found that ATR phosphorylation was accompanied by microtubule-associated protein 1 light chain 3B II (LC3B-II) expression during the early phases following UV irradiation, which is a well-established inducer of ATR. Knocking down ATR further attenuated the reduction in LC3B-II at early stages in response to UV treatment. Despite the potential role of ATR in autophagic response, reduced ATR expression does not affect autophagy induction during late phases (24 and 48 h after UV treatment). The result is consistent with the reduced ATR phosphorylation at the same time points and suggests that autophagic response at this stage is activated via a distinct pathway. In conclusion, this study demonstrated that autophagy acts as a cytoprotective mechanism against UV-induced apoptosis and that autophagy induction accompanied with apoptosis at late stages is independent of ATR activation.

## 1. Introduction

Macroautophagy (referred to as “autophagy” from this point onward) is an evolutionarily conserved process in which long-lived proteins and intact organelles are engulfed within double-membraned vacuoles (autophagosomes) and then degraded after the vacuoles fuse with lysosomes (autophagolysosomes or autolysosomes). At the end of the process, the degradation products are released into the cytosol and reutilized for metabolism. The molecular machinery of autophagy is tightly regulated by diverse signaling pathways that are activated in response to various stimuli. Under physiological conditions, autophagy occurs at a basal level to maintain homeostasis. Nevertheless, autophagy can result in cell death if it is massively activated under certain stresses, an event which is referred to as programmed cell death type II [1]. Further evidence has indicated that autophagy also functions as a cytoprotective response against various types of cellular stress by providing the cell with metabolic substrates.

Recently, genotoxic stress-induced autophagy has been reported to result in both autophagic cell death and cytoprotection in response to various stimuli in multiple cell types [2–5]. Although numerous factors can lead to DNA damage, including reactive metabolic byproducts and environmental mutagens, previous reports have focused mainly on autophagy triggered by DNA damage resulting from chemical substances, ionizing radiation (IR), and reactive oxygen species that induce alterations in DNA structure. DNA damages caused by IR results in DNA double-strand breaks, which induce the activation of ataxia-telangiectasia mutated (ATM) and downstream signaling pathways [6].

Ataxia-telangiectasia mutated (ATM) and ATM and RAD3-related (ATR) are two of the major regulators of the cell’s DNA-damage response. These two proteins respond to various types of genotoxic stress by promoting cell cycle arrest, DNA repair, and apoptosis [7]. ATM and ATR are large kinases with significant sequence homology; therefore, they share many biochemical and functional similarities, and they phosphorylate many of the same substrates. In contrast to ATM, ATR is essential for survival because ATR-deficient mice [8,9] and human cells [10] are not viable. ATM responds primarily to double-stranded breaks in DNA, while ATR also reacts to damage caused by ultraviolet (UV) light or stalled replication forks [11,12]. Despite a previous study establishing a link between UV irradiation and autophagy induction [13], the biological meaning and signalling pathways involved in this process are still unknown.

In this study, we aimed to investigate whether UV radiation induces DNA damage accompanied by autophagy and the biological meaning of autophagy itself. In our previous study, we found that knocking down ATM attenuated the autophagy induced by BO-1051, an *N*-mustard linked with a DNA-affinic molecule [14]. Other studies have shown that ATM is involved in genotoxic stress-induced autophagy and that it integrates the DNA damage signaling pathways with cytoprotective events [5,15]. Because UV radiation can induce ATR activation and ATM shares both a high level of similarity and overlapping subtrates with ATR, it is possible that ATR may also plays a role in regulating the UV-induced autophagic response. We found that autophagy is induced in response to UV radiation. When autophagy was inhibited by beclin-1 and Atg5 knockdown, apoptosis increased, which suggests that autophagy acts as cytoprotective mechanism against UV-induced DNA damage. To our surprise, ATR phosphorylation was only correlated with early LC3B-II expression, which indicates that the protective autophagy at late stages when apoptosis occurred is activated through an ATR-independent pathway.

## 2. Results and Discussion

### 2.1. Autophagy Was Induced after UV Irradiation

**Figure 1:**
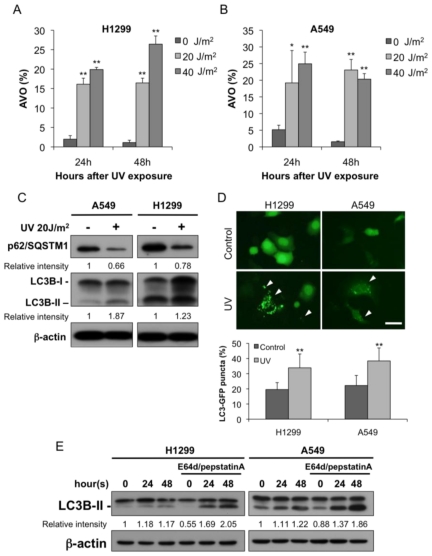
Induction of autophagy following the UV treatment. The (**A**) H1299 and (**B**) A549 cells were exposed to UV radiation (0, 20, or 40 J/m^2^), and collected 24 or 48 h later. The cells were stained with acridine orange and subsequently analyzed by flow cytometry; (**C**) The A549 and H1299 cells were treated with UV radiation (20 J/m^2^) and incubated for 24 h. The protein extracted from the harvested cells was resolved by SDS-PAGE, transferred to PVDF and probed with the indicated antibodies; (**D**) Punctuate signal of LC3-GFP in UV-treated H1299 and A549 cells. Quantification of cells expressing LC3-aggregation 24 h after UV treatment (20 J/m^2^). The percentage of LC3 aggregated cells was quantitated by counting the number of the cells showing the punctate pattern of LC3-GFP in GFP-positive cells. The arrowheads indicate cells with GFP puncta. Bar = 50 μm; (**E**) The H1299 and A549 cells were exposed to UV radiation (20 J/m^2^) and incubated for the indicated times. The extracted proteins were then immunoblotted with the indicated antibodies. E64d (10 μg/mL) and pepstatin A (10 μg/mL) were added to the medium where indicated. The detected bands were quantified using the ImageJ (NCBI) software and normalized to an internal control. The data represent the mean ± SD for at least three independent experiments. * *p* < 0.05 and ** *p* < 0.01 are statistically significant when compared to the untreated controls.

First, we examined whether UV irradiation could induce autophagy in the A549 and H1299 cells. An increase in the number of AVOs was detected in the A549 and H1299 cells 24 and 48 h after UV irradiation (Figure 1A,B). We also examined the known markers of autophagy and observed an increase in LC3B-II and a reduction in p62/SQSTM1 in both cell lines (Figure 1C). Autophagosome formation can be monitored by translocation of the autophagosome protein LC3B from the cytosol (diffuse distribution) to newly formed autophagosomes, which appear as cytoplasmic puncta. We established the H1299 and A549 clones transfected with LC3 fused to green fluorescent protein (LC3-GFP). Under a fluorescence microscope, LC3-GFP transfected cells showed diffuse distribution of green fluorescence in the control groups. In contrast, UV exposure increased a punctate pattern indicating that LC3 is recruited to the membrane of autophagosome. After quantifying the induction of cells expressing LC3 aggregation, a significant induction of LC3-GFP puncta formation was observed in both H1299 and A549 cells treated with UV (Figure 1D).

The accumulation of autophagosomes and autophagolysosomes after the UV treatment could involve either enhanced autophagic sequestration or reduced degradation of autophagic material [16]. To discriminate between these two possibilities, we assessed UV-induced autophagic vacuolization by adding two lysosomal protease inhibitors, E64d and pepstatin A. As shown in (Figure 1E), the inclusion of the two inhibitors further enhanced the UV-induced LC3B-II accumulation. Accordingly, the UV irradiation induced autophagic activity (also called on-rate autophagic flux) in both the A549 and the H1299 cells.

### 2.2. Autophagy Inhibition Enhances UV-Induced Cell Death

**Figure 2:**
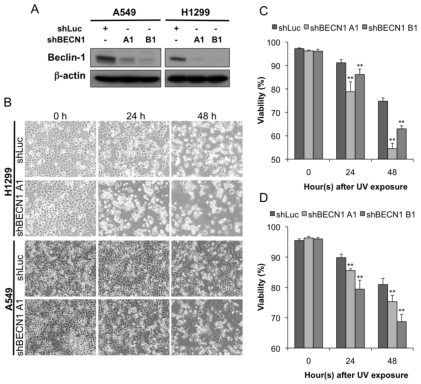
Down-regulation of the autophagy protein beclin-1 accelerates UV-induced cell death. (**A**) The knockdown efficiency of two shRNAs specific for beclin-1 was evaluated by immunoblotting; (**B**) The cells overexpressing either shLuc or shBECN1 A1 were exposed to UV radiation (20 J/m^2^), incubated for the indicated times, and examined by imaging analysis; The (**C**) H1299 and (**D**) A549 cells overexpressing either shLuc or shBECN1 (A1 and B1) were exposed to UV radiation (20 J/m^2^). After incubation for the indicated time, the cells were harvested and double-stained with annexin V-FITC and PI. The cell survival (for the PI-negative and annexin V-negative population) was determined by flow cytometry. The data are representative of three independent experiments and are shown as the mean ± SD. ** *p* < 0.01 is considered statistically significant when compared to the shLuc control during the same time course after UV exposure.

While it has been demonstrated that UV can induce autophagy, its role in the UV-induced cell death that results from ATR activation is undetermined. Autophagy has been found to cause either cell death or cytoprotection in response to various stimuli [17]. Because UV radiation is known to result in cell death by apoptosis [18], it is more likely that autophagy acts as a cytoprotective mechanism against the cellular stress caused by UV exposure. To further investigate the biological significance of UV-induced autophagy, we applied the shRNA gene-silencing method to knock down beclin-1, which plays a key role in the formation of autophagosomes [19]. Our data confirmed the knockdown efficiency of the shRNAs (Figure 2A). Cell survival was observed microscopically (Figure 2B) and determined by flow cytometry using PI staining. Cell viability was significantly lower in the shBECN1 group (both A1 and B1) than in the shLuc control group (*p* < 0.05; Figure 2C,D). Therefore, autophagy appears to have a cytoprotective effect in UV-induced cell death.

**Figure 3:**
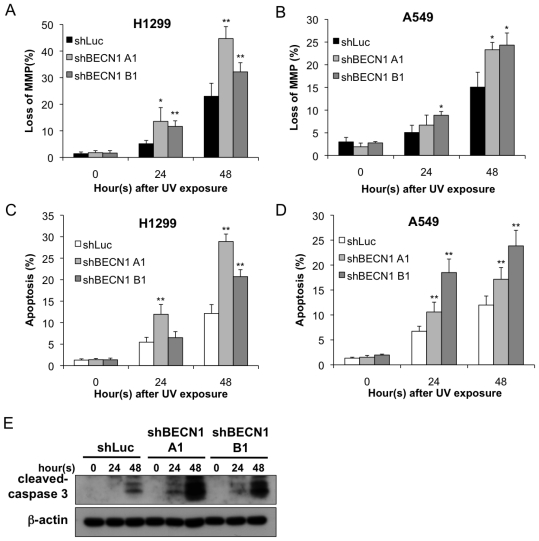
Inhibition of autophagy through knocking down beclin-1 enhanced the loss of MMP and apoptosis following UV exposure. The (**A** and **C**) H1299 and (**B** and **D**) A549 cells overexpressing shLuc or shBECN1 were exposed to UV radiation (20 J/m^2^). The cells were harvested after the indicated time and were stained with JC-1 or annexin V-FITC and PI as detailed in the Materials and Methods section. The data are representative of three independent experiments and are shown as the mean ± SD. * *p* < 0.05 and ** *p* < 0.01; both are considered statistically significant when compared to the shLuc controls at 24 or 48 h after UV exposure; (**E**) The cell lysates were analyzed by immunoblotting using the indicated antibodies.

We next investigated whether autophagy inhibition-induced cell death is mediated by an augmentation of apoptosis. As shown in Figure 3A,B, the loss of mitochondrial membrane potential (MMP) was enhanced in both shBECN1 groups. The quantitative results showed a significant increase in the percentage of cells that lost their MMP after UV exposure (*p* < 0.05; Figure 3A,B). A similar result was obtained by the annexin V staining assay, in which the shBECN1-knockdown cells showed an increased population of apoptotic cells (Figure 3C,D). Furthermore, the expression of cleaved caspase-3 increased when autophagy was inhibited by knocking down Beclin-1 (Figure 3E).

**Figure 4:**
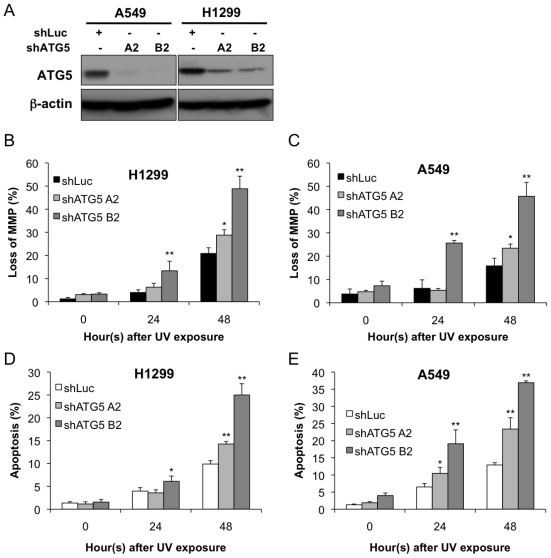
Inhibition of autophagy through knocking down Atg5 augments UV-induced loss of MMP and apoptosis. (**A**) The knockdown efficiency of two shRNAs specific for Atg5 was evaluated by immunoblotting; The (**B** and **D**) H1299 and (**C** and **E**) A549 cells overexpressing shLuc or shAtg5 were exposed to UV radiation (20 J/m^2^). The cells were harvested after the indicated time and were stained with JC-1 or annexin V-FITC and PI and subjected to flow cytometric analysis. The data are representative of three independent experiments and are shown as the mean ± SD. * *p* < 0.05 and ** *p* < 0.01; both are considered statistically significant when compared to the shLuc control during the same time course after UV exposure.

In order to confirm that survival is broadly due to a defect in autophagy rather than a phenotype attributable to an individual ATG, we applied another shRNA targeting Atg5, which is an essential protein for autophagosome formation [20]. In Figure 4A, the knockdown efficiency of shRNAs targeting Atg5 was confirmed. The H1299 and A549 cells overexpressing shAtg5 (A2 and B2) showed increased loss of MMP and enhanced apoptosis as compared with shLuc control group (*p* < 0.05; Figure 4B–E). Collectively, these results indicate that autophagy provides cytoprotection against UV-induced apoptosis.

### 2.3. ATR Activation Parallels the Induction of Autophagy at Early Stages Following UV Treatment

**Figure 5:**
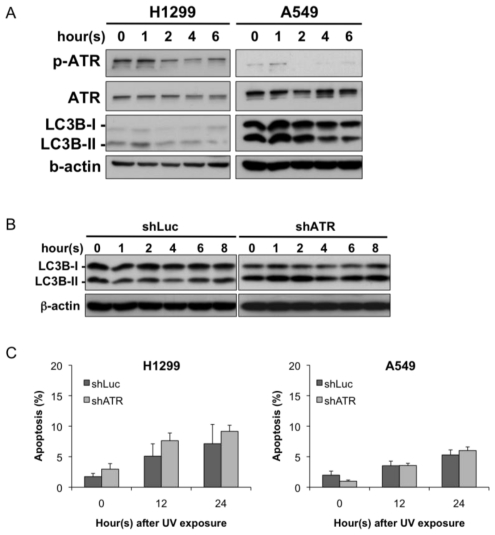
The early wave of LC3B-II is correlated with ATR phosphorylation. (**A**) UV radiation induces ATR phosphorylation and alteration of LC3B expression. The A549 and H1299 cells were exposed to UV radiation (20 J/m^2^) and incubated for the indicated times. The protein extracts were then subjected to immunoblotting using the indicated antibodies; (**B**) The A549 cells overexpressing shLuc or shATRs were exposed to UV radiation (20 J/m^2^) and immunoblotted with the indicated antibodies; (**C**) The H1299 and A549 cells overexpressing shLuc or shATR were exposed to UV radiation (20 J/m^2^). The cells were harvested after the indicated time and were stained with annexin V-FITC and PI and subjected to flow cytometric analysis. The data are representative of three independent experiments and are shown as the mean ± SD. No significant difference was found when compared to the shLuc control during the same time course after UV exposure.

To study the role of ATR in UV-induced autophagy, we examined the phosphorylation of Chk1, a downstream substrate of ATR. The levels of phosphorylated Chk1 quickly rose after UV exposure and then diminished over time (Figure S1). Therefore, we focused on the early time points (within 6 h) after UV exposure to study the relationship between ATR and autophagy activation. As shown in Figure 5A, phosphorylated ATR increased within 1 h of the UV treatment and then quickly diminished after its activation. To our surprise, the conversion of LC3B-I to LC3B-II increased only slightly during the peak of ATR activation, and it also diminished quickly after the UV exposure. Quantification of the phosphorylated ATR and LC3B-II expression level revealed that these two proteins have a positive correlation (Figure S2), suggesting that UV treatment leads to simultaneous activation of ATR and alteration of the LC3B-II conversion within 6 h after UV treatment.

While the early wave of LC3-II induction correlates with ATR phosphorylation, we further knocked down ATR expression by shRNA (Figure S3) to evaluate the effect of ATR on LC3B conversion at the early stages following UV treatment. The knockdown efficiency was limited and a small amount of ATR was retained because ATR is required for viability in human cells [10]. The effect of the ATR knockdown on endogenous LC3B expression was evaluated (Figure S4). There was no significant alteration of LC3B expression in two cell lines. Nevertheless, when the cells were exposed to UV radiation, the originally decrease in LC3B-II conversion was attenuated when compared to the shLuc controls (Figure 5B). These data indicate that ATR plays a role in UV-induced early autophagic response. Nevertheless, this early wave of LC3-II and ATR phosphorylation did not impact survival in the first 12–24 h following UV treatment (Figure 5C).

### 2.4. Autophagy Induction at Late Stages Is Independent of ATR

**Figure 6:**
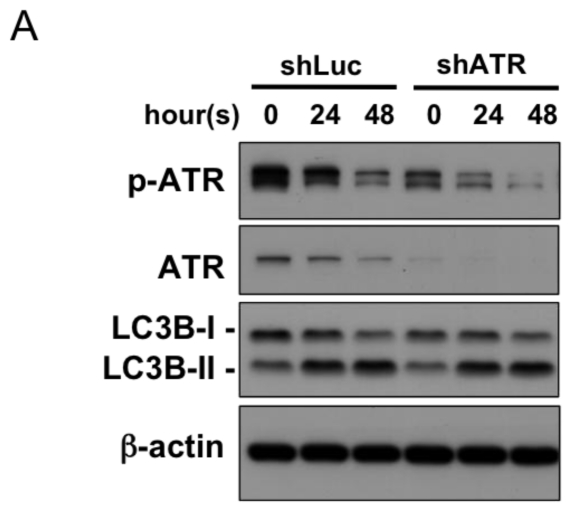

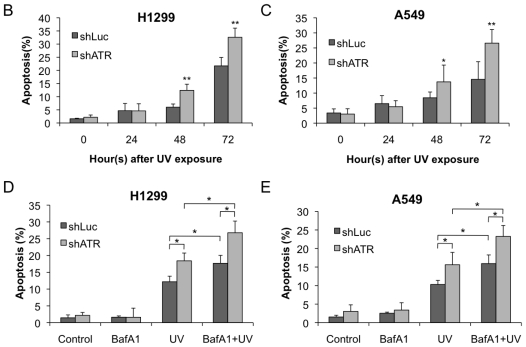
Autophagy provides cytoprotection in ATR-deficient cells. (**A**) The A549 cells overexpressing shLuc or shATR were exposed to UV radiation (20 J/m^2^). The protein extracts from the cells overexpressing shLuc or shATR were immunoblotted with the indicated antibodies; (**B** and **C**) The H1299 and A549 cells overexpressing shLuc or shATRs were exposed to UV radiation (20 J/m^2^). The cells harvested after the indicated time were stained with annexin V-FITC and PI and subjected to flow cytometric analysis; (**D** and **E**) The H1299 and A549 cells overexpressing shLuc or shATRs were exposed to UV radiation (20 J/m^2^). BafA1 (10 nM) was added to the medium 6 h before UV exposure. The apoptosis was assessed using annexin V staining. The data are representative of three independent experiments and are shown as the mean ± SD. * *p* < 0.05 and ** *p* < 0.01; both are considered statistically significant when compared to the shLuc control during the same time course after UV exposure.

While the early wave of LC3B conversion and ATR phosphorylation does not affect survival of the H1299 and A549 cells following UV treatment. We would like to know whether ATR is necessary for autophagy at a later time point (24 and 48 h after UV treatment). As shown in Figure 6A, ATR phosphorylation decreased 24 and 48 h after UV exposure in the shLuc control group. Consistent with the result, knockdown of ATR did not influence the induction of autophagy at these time points (24 and 48 h). Therefore, ATR should not be involved in autophagic response at 24 and 48 h.

On the other hand, previous studies have reported that ATR inhibition promoted apoptosis induced by UV [21,22]. We explored whether autophagy still provides cytoprotective effects in cells with lower ATR expression. Our data confirmed that apoptosis was significantly enhanced in the H1299 and A549 cells overexpressing shATR after UV exposure (Figure 6B,C). We applied an autophagy inhibitor, bafilomycin A1 (BafA1), to investigate the role of autophagy in ATR-deficient cells. BafA1 is an inhibitor of vacuolar ATPase (V-ATPase) and prevents the fusion between lysosomes and autophagosomes. In both the H1299 and A549 cells, autophagy inhibition using BafA1 enhanced apoptosis in cells overexpressing shLuc or shATR (Figure 6D,E) suggesting that autophagy provides protection that is independent of ATR existence.

## 3. Discussion

While autophagy can be a form of programmed cell death when activated extensively, it is widely accepted that in most circumstances, autophagy acts as a cytoprotective mechanism against various types of cellular stress [23]. Previous research has described the effects of UV irradiation on autophagy induction without demonstrating the biological meaning of autophagy itself [13]. However, few studies have examined the effects of autophagy on cell viability in response to UV irradiation without demonstrating whether autophagy was induced or not [24,25]. In this study, we both demonstrated that the UV irradiation induced on-rate autophagy and found that autophagy acted as a cytoprotective mechanism in both the A549 and H1299 cell lines because knockdown of the two autophagy proteins, Beclin-1 and ATG5, enhanced the UV-induced cell death. Nevertheless, the role of autophaogy in preventing cell death is controversial. One previous study has shown that the loss of Atg5 makes cells more resistant to various doses of UV treatment [25,26]; only a tetrazolium salt colorimetric assay was used to evaluate cell viability, however, so this result may not accurately represent the actual overall cell death percentage. By contrast, beclin-1-deficient embryonic stem (ES) cells showed no differences in cell death induced by either UV or serum withdrawal when compared to a wild type control [24]. It has also been reported that a beclin-1 knockdown can inhibit viability in Chang cells [27]. Therefore, the role of autophagy in determining cell fate is likely cell type-dependent manner and further investigation is required to explore the underlying mechanisms.

Autophagy is known to be regulated by several different pathways. Repression of the mTOR pathway can activate autophagy in all eukaryotic cells, while the Erk1/2 and p38-associated pathways may only be activated under certain conditions in specific cell types [28]. A previous study has suggested that ATM is capable of regulating autophagy by repressing mTOR via the LKB1-AMPK-TSC2 pathway [15]. It is possible that ATR utilizes the same signal-transduction pathway. The underlying mechanism by which ATR signals to downstream signaling cascades to modulate autophagy is still unclear.

ATM and ATR are members of the phosphoinositide 3-kinase-related kinases (PIKK) superfamily. In response to DNA damage, ATM and ATR mediate a variety of downstream pathways that modulate DNA repair, cell cycle checkpoints, and apoptosis [18,29]. Despite these two kinases sharing many similarities, such as biochemical functions and most of their downstream targets, there are some differences as well. It is well established that ATM senses primarily double-strand breaks (DSBs), while ATR also responds to UV irradiation and stalled replication forks [29,30]. In contrast to ATM, moreover, ATR is required for viability [9], which indicates that ATR is essential for normal cell growth. Because ATM has been shown to participate in DNA damage-induced autophagy [5,14,15], the possibility exists that ATR may also participate in regulating autophagy. In our study, we showed that UV irradiation can induce ATR activation, which is positively correlated with LC3B expression (Figure 5) at early stages. Although the induction of LC3B-II correlated with the pattern of ATR activation, LC3B-II was soon diminished after the inactivation of ATR. The induction of autophagy that occurs 24 or 48 h after UV treatment seems to be independent of ATR activation, possibly indicating that another mechanism mediates the late phase of autophagic induction and exerts survival effects on cells exposed to UV irradiation. This rescue effect comes, at least in part, from providing metabolic substrates because the addition of methyl pyruvate rescued some of the cells from apoptosis (data not shown). To our knowledge, ours is the first study providing direct evidence demonstrating the relationship between ATR and autophagy in a human cell model. We both demonstrated the role of ATR in regulating autophagy and provided a association between UV-induced apoptosis and autophagy.

In previous studies, UV irradiation has resulted in the formation of DNA photoproducts, mainly *cis*-*syn* cyclobutane pyrimidine dimers (CPDs) and pyrimidine (6-4) pyrimidone photoproducts (6-4PPs). ATR is the major sensor of these UV-induced DNA lesions. Nevertheless, additional evidence has shown that ATM can be phosphorylated by ATR after UV irradiation via a DSBs formation-independent mechanism [31]. Because ATM is involved in DNA damage-induced autophagy, the possibility that ATM mediates UV-induced autophagy also arises. ATR may transmit a signal to ATM and its downstream targets resulting in autophagy induction. However, it is also been found that ATR can be recruited to the site of DSBs and that this recruitment is indirectly mediated by ATM [32,33]. The interaction between ATM and ATR provides clues to constructing the circuit that results in DNA damage-induced autophagy. The evidence mentioned above does not raise doubt about role of ATR in UV-induced autophagy but rather provides a linkage between ATM and ATR and their significant roles in DNA damage-induced metabolic regulation.

## 4. Materials and Methods

### 4.1. Materials

Acridine orange, E64d, pepstatin A, and Bafilomycin A1 were purchased from Sigma Chemical Co. (St. Louis, MO, USA).

### 4.2. Cell Culture and UV Irradiation

A549 and H1299 cells were cultured in Dulbecco’s Modified Eagle’s Medium (GIBCO, Grand Island, NY, USA) with 10% fetal bovine serum (GIBCO), 100 U/mL penicillin, and 100 mg/mL streptomycin (GIBCO) under standard culture conditions (37 °C, 95% humidified air, and 5% CO_2_). The cells were plated in 6-cm dishes 24 h prior to the UV treatment. The cells were irradiated at the indicated doses with a UV lamp (254 nm) and were then incubated at 37 °C for the indicated time.

### 4.3. Detection of Acidic Vesicular Organelles (AVOs) with Acridine Orange

To quantify the development of AVOs in the UV-treated cells, they were stained with acridine orange, and the intensity of the red fluorescence was measured by flow cytometry. Green (510–530 nm) and red (>650 nm) fluorescent emissions from 10,000 cells illuminated with a blue (488 nm) excitation light were measured with a FACSCalibur from Becton Dickinson (San Jose, CA, USA) using the CellQuest software.

### 4.4. Immunoblotting

The harvested cells were pelleted by centrifugation, washed with PBS, and lysed with M-PER buffer (Pierce, Rockford, IL, USA). The protein content was measured with a protein assay kit (Bio-Rad Laboratories, Hercules, CA, USA). A 20-μg aliquot of the total protein was separated by SDS/PAGE (10% or 12% gels) and transferred to PVDF membranes (Millipore Corporation, Bedford, MA, USA) for immunological detection of the proteins. The blots were probed using antibodies against p62/SQSTM1 (Progen Biotechnik, Heidelberg, Germany), LC3B, Beclin-1, cleaved caspase-3, p-ATR, ATR, p-Chk1, Chk1, p-AMPKa, AMPKa (Cell Signaling Technology, Beverly, MA, USA), and β-actin (Millipore Corporation, Bedford, MA, USA) according to the manufacture’s instructions. To quantify the bands obtained from the immunoblot analysis, we applied an ImageJ (NCBI) software-based analysis. The phosphorylated ATR and LC3B-II levels were normalized to an internal control and displayed as the relative expression.

### 4.5. Apoptosis Assay

FITC-conjugated annexin V was used to detect the presence of apoptosis. The cells were seeded in 6-cm dishes one day prior to the UV exposure. After the UV irradiation for the indicated doses and times, the cells were harvested and stained with annexin V-FITC and PI (BD Falcon, Bedford, MA, USA) according to the manufacturer’s instructions. The resulting fluorescence was detected by flow cytometry.

### 4.6. Mitochondrial Membrane Potential Analysis

The loss of mitochondrial membrane potential was quantitatively determined by flow cytometry using JC-1 staining; this assay was performed according to the manufacturer’s instructions (Molecular Probes, Invitrogen Life Technologies, Carlsbad, CA, USA). Briefly, the cells were trypsinized, washed with PBS, and resuspended in PBS at a concentration of 1 × 10^6^ cells/mL. The cells were then stained with 2.5 μL of JC-1 (1 mg/mL) and incubated in the dark at 37 °C for 30 min. The positive cells were subsequently detected by FACSCalibur flow cytometer with CellQuest analysis software (Becton Dickinson).

### 4.7. shRNA Expression Constructs and Lentiviral Transduction

Stable ablation of beclin-1 in the A549 and H1299 cell lines was accomplished using small hairpin RNA (shRNA) probes TRCN0000033549 (shBECN1 A1) and TRCN0000033550 (shBECN1 B1) for the Homo sapiens beclin-1 gene (BECN1; NM_003766). The A549 and H1299 cells stably expressing shATG5 were created using the shRNA probe TRCN0000151474 (shATG5 A2) and TRCN0000151963 (shATG5 B2) for the Homo sapiens ATG5 gene (NM_004849). The cells stably expressing shATR were created using the shRNA probe TRCN0000219647 (shATR) for the Homo sapiens ATR gene (NM_001184). The control cells stably expressed shLuc (pLKO.1-shLuc). The cells were infected with shRNA lentiviruses that were generated using a three-plasmid-based lentiviral system (all the plasmids are available from The RNAi Consortium (TRC)). Lentiviral production was performed by transfection of 293T cells at 5 × 10^6^ cells per 10-cm plate using Lipofectamine 2000 (LF2000, Invitrogen Life Technologies, Carlsbad, CA USA). The supernatants were collected 48 h after transfection and subsequently filtered. The subconfluent cells were infected with lentiviruses in the presence of 8 μg/mL Polybrene (Sigma). The infected cells were selected with puromycin (2 μg/mL) until all of the uninfected control cells were dead. Immunoblotting was used to confirm the knockdown efficiency of shBECN1, shATG5, and shATR.

### 4.8. LC3-GFP Puncta

The LC3-GFP expression vector was kindly gifted by Dr. Kuo-Wei Chang (National Yang-Ming University, Taipei, Taiwan). The H1299 and A549 cells expressing LC3-GFP were established. Transfection was performed on 6-well plate with 2 μg plasmid DNA/well with jetPEI transfection reagent (Polyplus-transfection France) according to the manufacturer’s instructions. At 48 h after transfection, cells were exposed to UV irradiation and were analyzed by fluorescence microscopy at indicated times. The percentage of LC3 aggregated cells was quantitated by counting the number of the cells showing the punctate pattern of LC3-GFP in GFP-positive cells.

### 4.9. Data Analysis

Data are expressed as the mean ± SD from at least three independent experiments. The statistical analysis was performed using the Student’s *t*-test. Differences were considered significant when *p* < 0.05.

## 5. Conclusions

We have demonstrated that UV irradiation induces on-rate autophagy in the A549 and H1299 cells. Autophagy inhibition through Beclin-1 and ATG5 knockdown enhances UV-induced apoptosis. Furthermore, in the early phases of the UV-induced cellular response, LC3B expression occurs in parallel to both ATR activation and diminished expression. The ATR knockdown makes cells less responsive to UV-triggered autophagy at early stages following UV treatment. Although ATR phosphorylation correlates with the early wave of LC3B conversion, autophagy provides ATR-independent cytoprotection against apoptosis at late stages following UV irradiation. Therefore, our findings demonstrate the significance of autophagy in response to UV-induced DNA damage and apoptotic cell death.

## Supplementary Materials
