# Supplementary material for: Targeting Protective Autophagy Exacerbates UV-Triggered Apoptotic Cell Death
Source: Int J Mol Sci. 2012 Jan 20;13(1):1209–24. doi: 10.3390/ijms13011209 (PMC3269747; doi:10.3390/ijms13011209)
Supplement: Supplementary file 1 [file ijms-13-01209-s001.pdf]

Figure S1. The time course of Chk1 phosphorylation after UV treatment. The H1299 and A549 cells were exposed to UV radiation (20 J/m^2). The cells were harvested after incubation for the indicated times. The protein extracts were subjected to immunoblotting with the indicated antibodies.

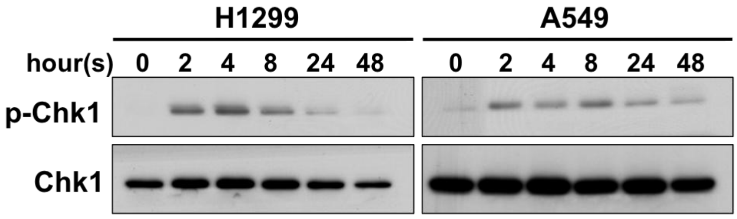

Figure S2. The quantification of the phosphorylated ATR and LC3B-II expression in the A549 cells is shown. The detected bands were quantified using the ImageJ (NCBI) software and normalized to an internal control. The data are representative of three independent experiments and are shown as the mean ± SD.

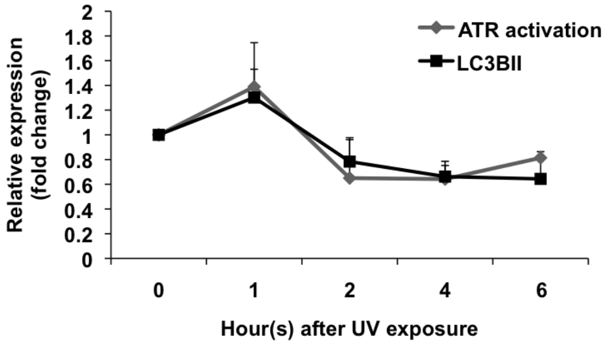

Figure S3. The knockdown efficiency of shRNA specific for ATR was evaluated. The protein extracts from the cells overexpressing shLuc or shATR were immunoblotted with the indicated antibodies.

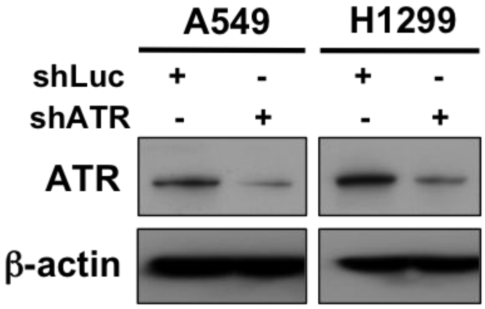

Figure S4. ATR does not affect endogenous LC3B expression. The protein extracts from the cells overexpressing shLuc or shATR were immunoblotted with the indicated antibodies to evaluate the effects of the ATR knockdown on LC3B-II conversion.

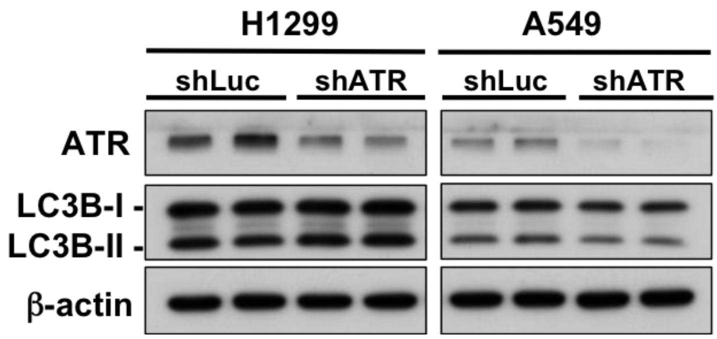

© 2012 by the authors; licensee MDPI, Basel, Switzerland. This article is an open access article distributed under the terms and conditions of the Creative Commons Attribution license (http://creativecommons.org/licenses/by/3.0/).
